# Redundancy-aware unsupervised ranking based on game theory: Ranking pathways in collections of gene sets

**DOI:** 10.1371/journal.pone.0282699

**Published:** 2023-03-09

**Authors:** Chiara Balestra, Carlo Maj, Emmanuel Müller, Andreas Mayr

**Affiliations:** 1 Department of Computer Science, TU Dortmund, Dortmund, Germany; 2 Department of Medical Biometry, Informatics and Epidemiology (IMBIE), University Hospital Bonn, Bonn, Germany; 3 Institute for Genomic Statistics and Bioinformatics IGSB, University Hospital Bonn, Bonn, Germany; 4 Centre for Human Genetics, University of Marburg, Marburg, Germany; Universita degli Studi di Roma Tor Vergata, ITALY

## Abstract

In Genetics, gene sets are grouped in collections concerning their biological function. This often leads to high-dimensional, overlapping, and redundant families of sets, thus precluding a straightforward interpretation of their biological meaning. In Data Mining, it is often argued that techniques to reduce the dimensionality of data could increase the maneuverability and consequently the interpretability of large data. In the past years, moreover, we witnessed an increasing consciousness of the importance of understanding data and interpretable models in the machine learning and bioinformatics communities. On the one hand, there exist techniques aiming to aggregate overlapping gene sets to create larger pathways. While these methods could partly solve the large size of the collections’ problem, modifying biological pathways is hardly justifiable in this biological context. On the other hand, the representation methods to increase interpretability of collections of gene sets that have been proposed so far have proved to be insufficient. Inspired by this Bioinformatics context, we propose a method to rank sets within a family of sets based on the distribution of the singletons and their size. We obtain sets’ importance scores by computing Shapley values; Making use of microarray games, we do not incur the typical exponential computational complexity. Moreover, we address the challenge of constructing redundancy-aware rankings where, in our case, redundancy is a quantity proportional to the size of intersections among the sets in the collections. We use the obtained rankings to reduce the dimension of the families, therefore showing lower redundancy among sets while still preserving a high coverage of their elements. We finally evaluate our approach for collections of gene sets and apply Gene Sets Enrichment Analysis techniques to the now smaller collections: As expected, the unsupervised nature of the proposed rankings allows for unremarkable differences in the number of significant gene sets for specific phenotypic traits. In contrast, the number of performed statistical tests can be drastically reduced. The proposed rankings show a practical utility in bioinformatics to increase interpretability of the collections of gene sets and a step forward to include redundancy-awareness into Shapley values computations.

## 1 Introduction

When working with collections of gene sets, one of the main challenges is the sheer size and low interpretability of the many gene sets belonging to the same collection. We refer to gene sets or *pathways* as sets of genes deriving from a biological classification of genes with respect to chemical or biological functions; these are grouped in variably sized collections based on some prior biological or chemical functions [1]. Following this grouping, scarcely interpretable collections whose size ranges from hundreds to several thousand of partly overlapping gene sets exist. The main challenges to be faced are then the low chances for the human eye to recollect the information conveyed by the collections and the pathways in them. The collections are often used for Gene Set Analysis (GSA), i.e., a variety of methods that assess the enrichment of genes in different gene sets concerning a phenotype. Many of these methods rely on multiple statistical tests intending to identify biological mechanisms potentially associated with a phenotype; statistical tests, such as Fisher Exact Tests, are performed assuming that a list of target genes is relevant for the analyzed trait. A statistical test has to be performed for each of the pathways in the collection; thus, the multiple hypothesis correction are stricter the larger the size of the family of gene sets.

We introduce a new method based on Cooperative Game Theory (CGT) to reduce a priori (independently from a particular phenotype) the number of gene sets in the collections. Introduced in CG by Shapley [2], Shapley values allow fairly allocating common resources in games among players. In the recent years, CGT found place in supervised feature selection where the scores obtained by Shapley values help discriminate among relevant and non-relevant features for the task at hand. Shapley values derive their success from the flexible and non-demanding definition of the *value function*, i.e., the allocation function of the resources in the game, making them an easily applicable tool in various contexts, e.g., feature selection, interpretable machine learning, allocation of resources, and many others [3–5].

In our framework, we aim to reduce the size of collections of gene sets in a more general setting: we assign importance scores to sets within families of sets, reformulating the problem in terms of allocating resources in a cooperative game. Using the Shapley value allocation of resources within the family of sets, we assign importance scores to sets depending on how their elements are distributed throughout the whole family. The scores are used to rank the sets and reduce the size of the families. When applied to the collection of gene sets, the derived selection is independent of the GSA method and the chosen phenotypic traits; thus, we classify our approach as an *unsupervised selection method*. We show that the Shapley values are positively correlated with the size of the sets and that they are unaware of intersections among them; hence, redundant (i.e., overlapping) sets are allocated by Shapley values in similar ranking positions. We address this issue by adding a pruning criterion to the Shapley values to get a new ranking of sets showing no correlation with the sets’ sizes and low overlap among similarly ranked sets. To the best of our knowledge, this is the first time that an unsupervised redundancy-aware ranking of sets has appeared in the literature. We use the collections of gene sets as an application environment of the proposed theoretical method; the consequent ranking and selection of pathways are characterized by a considerable reduction of the overlaps among similarly ranked gene sets and high coverage of the genes in the collections. Furthermore, we illustrate that our penalized Shapley values affect the correlation between the size of gene sets and their position in the rankings, although not directly meant to solve this issue. The rankings show excellent behavior in selecting gene sets using Shapley values approximating the solution of a min-max-max problem, i.e., minimizing the overlap and maximizing both the importance scores assigned by Shapley values and the coverage of genes. Finally, the results suggest that the switch to smaller collections of gene sets does not affect the coverage of the genes.

## 2 Related work

The CGT framework led to developments in various contexts and applications in the computer science community. In particular, Shapley values have been extended to supervised, semi-supervised and unsupervised feature selection [5–7], networks and security [8] and explainable machine learning [4]. A recent article by Rozemberczki et al. [3] summarizes most of the applications in machine learning literature. Moreover, importance scores based on Shapley values have been adapted to study the interaction among genetic and phenotypic characteristics for gene sets prioritization analysis [9, 10]. As a major drawback, Shapley values’ exact computation requires 2^*N*^ evaluations of a value function where *N* is the number of players. The computational complexity of these scores makes their application unfeasible as soon as the number of players increases. As a response, many approximation techniques of Shapley values appeared in machine learning literature [8, 11], based on Monte Carlo sampling procedures or stratification of the players. In bioinformatics applications, it has been argued that the introduction of microarray games [12] reduces the computational challenges of exact Shapley values’ computation to polynomial time under the assumption that the game can be written using only binary relationships (i.e., *anomalous* vs. *normal*, *in* vs. *not in* among others). Sun et al. [13] made use of Shapley values within microarray games to rank genes by their relevance with respect to the individual genes’ synergistic influence in a gene-to-gene interaction network.

As collections of gene sets are families of pathways [1] they are by nature arbitrary in size and contain partly overlapping gene sets. The sheer size of these collections consequently leads to high redundancy, i.e., pathways containing the same genes, and low interpretability. The overlap of pathways in collections of gene sets is a well-known problem: biologically, genes participate in numerous pathways representing various biological processes. Furthermore, the human understanding of the collections of gene sets is hindered by these overlaps and the high number of pathways in the collections: Some recent solutions proposed tools for visualizing redundancy among pathways, merging gene sets based on their similarity, and integrating gene sets into a non-redundant single and unified pathway [14–16]. Connected to this issue, Stoney et al. [17] point out the lack of agreement among the various collections of gene sets’ databases. Their method maximizes the gene coverage and does not alter the gene sets themselves from their original form. However, the authors handle redundancy among collections of gene sets in the different databases and *not* in the single collections. To the best of our knowledge, methods including information on the interactions among gene sets within the single collections have not yet appeared in the literature. Moreover, database redundancy and disagreement are not limited to collections of gene sets, e.g., [18, 19]. The impact of the choice of databases has been tackled for specific applications on cancer development in Mubeen et al. [20]. In contrast to other redundancy reduction methods, we aim to rank the original gene sets in the collections using Shapley values, thus relying on theoretical properties of fair allocation of resources. When comparing with the work by Stoney et al. [17], we point out the different goals of our rankings that maximize the coverage of genes while lowering redundancy *within* the collection of gene sets.

As already mentioned, one of the main applications of collections of gene sets is enrichment analyses, e.g., assessment of the potential over-representation or under-representation of the analyzed genes in specifically biologically annotated gene sets via Fisher tests, the GSEA algorithm [21, 22]; among the enrichment analysis tools, we recall Enrich [23–25], a web-based tool that provides various types of visualization summaries of collective functions of gene lists. The number of statistical hypothesis tests that need to be performed typically equals the number of pathways within the collection of gene sets. GSA approaches, therefore, test several times for genes belonging to multiple pathways. The correction for multiple testing naturally becomes a major challenge [26, 27] in high-dimensional collections. Different kinds of corrections for multiple testing are known to preserve the type-I error [28, 29] but often lead to a loss of power (type-II error) [30]. Among them, we recall the Bonferroni correction and the less conservative false discovery rate FDR [31, 32].

In the literature also appear alternative methods to identify gene sets associations [33, 34]. REVIGO [35] was proposed as a Web server to summarize the long unintelligible lists returned by statistical testing for enriched gene functional categories. Furthermore, in 2016, the first two unsupervised rankings for gene sets based on hypothesis testing appeared in Frost et al. [36].

## 3 Methods

In this section, we first introduce some basic notions of cooperative game theory and highlight some definitions we will use. A introductory toy example (the *glove game*) for these rather abstract concepts can be found in the S1 Appendix.

### 3.1 Cooperative game theory

L. Shapley first formalized Game Theory in 1951 [2]. A cooperative game is a pair (N,v) where N={1,…,N} is a finite set of players and $v:2^{N}\to R_{+}$ is the so-called *value function*. The value function *v* maps each subset T⊆F (usually referred to as *coalition*) of players to a real non-negative score *v*(*T*). A fundamental assumption is that the empty coalition has null value v(⌀)=0 while it is common to work with normalized games, i.e., v(N)=1.

The fair allocation of the value v(N) among the players in the game has been extensively studied: Fundamentally, each player can participate in the creation of 2^*N*−1^ coalitions where N=|N| and this is of use to create a fair allocation. Shapley values have been introduced to fairly compare the roles of players within a cooperative game. They represent one of the possibilities of dividing the payoff of N such that the amount of resources allocated to each of the player is fair concerning the player’s contribution in any possible coalition within the game. Moreover, it has been proved that Shapley values represent the only possible allocation of resources satisfying the *dummy*, *symmetry*, *null-player* and *efficiency* properties [2]. In particular, the efficiency property states that the sum of the Shapley values over the set of all players equals v(N).

The Shapley value of a player i∈N is the average of its *marginal contributions* *v*(*T* ∪ {*i*}) − *v*(*T*) across all possible coalitions T⊆N, i.e.,

$$\begin{array}{c} \phi_{i}\left(v\right)=\sum_{T\subseteq N\backslash \{i\}}\frac{(N-t-1)!\;t!}{(N-1)!}\cdot \left(v\left(T\cup \left\{i\right\}\right)-v\left(T\right)\right) \end{array}$$ (1)

where N=|N| and *t* = |*T*|.

In Eq (1), the value function needs to be computed 2^*N*^ times. Due to the exponential complexity, computational problems arise when the number of players increases. However, one particular class of games, the *Sum-Of-Unanimity Games* (SOUG) [2], admits a polynomial closed-form solution (see S1 Appendix for details) and microarray games are a special case of SOUG games.

### 3.2 Microarray games

Let us consider $F=\{P_{1},\ldots ,P_{N}\}$ a family of sets being the set of players. We denote with

$$\begin{array}{c} G=\left\{g\in P_{i}|P_{i}\in F\right\}=\underset{i\in \{1,\ldots ,n\}}{⋃}P_{i} \end{array}$$

the elements belonging to at least one set *P*_*i*_ and *M* = |*G*|. Starting from F and *G*, we build a binary matrix *B* ∈ {0, 1}^*N* × *M*^ where *B*_*ij*_ = 1 if *g*_*j*_ ∈ *P*_*i*_ and *B*_*ij*_ = 0 otherwise. Transposing the definition given by Moretti et al. [12], for each element *g*_*j*_ ∈ *G*, we look at the set of sets in which *g*_*j*_ is present; we call this set the support of *g*_*j*_ and write sp(*g*_*j*_). Formally, we obtain the support of *g*_*j*_ from the matrix *B*. the information on *g*_*j*_ is conveyed by the column *B*_*j*_ of *B*, and, by abuse of notation, we write sp(*g*_*j*_) or sp(*B*_*j*_) interchangeably. We define sp(*B*_*j*_) as the set

$$\begin{array}{ccc} \text{sp}(B_{j}) & = & \;\{P_{i}\in F|B_{ij}=1\}\\ & = & \;\{P_{i}\in F|g_{j}\in P_{i}\} \end{array}$$

i.e., the set of the sets containing *g*_*j*_. The *microarray game* is then defined as the cooperative game $\left(F,v^{*}\right)$ where, for each T⊆F,

$$\begin{array}{c} v^{*}\left(T\right)=\frac{\left|\Theta (T)\right|}{\left|G\right|}=\frac{\left|\{g_{j}\in G|\text{sp}\left(g_{j}\right)\subseteq T\;\text{and}\;\text{sp}\left(g_{j}\right)\neq ⌀\}\right|}{\left|G\right|}. \end{array}$$ (2)

Here the adapted value function v*(*T*) is the ratio of the number of genes’ supports that *T* contains and the number of elements in *G*. As |*G*| is fixed, we can simply say that v*(*T*) is proportional to the number of supports *T* contains; higher scores are achieved by sets covering the full distribution among sets of a high number of elements.

Following the approach by Sun et al. [13], the value function can be easily expressed in terms of a linear combination of unanimity games where each column is interpreted as a unanimity game. Using this formulation of the value function, the computation of Shapley values is reduced to polynomial time.

### 3.3 Computation of Shapley values and definition of the game

Shapley values are a common solution to assign fair scores to players within a cooperative game. However, they show an inherent problem: redundant players get similar scores, thus implying that they are ranked in close positions. The *glove game* example clearly illustrates the problem (see S1 Appendix). To solve this, we integrate a redundancy-awareness concept into Shapley values to rank players taking possible overlapping among them into account. In particular, the player ranked at the (*i* + 1)-th place should be the least overlapping possible with the first *i*-ranked players {*P*_1_, …, *P*_*i*_}. In order to achieve such a redundancy-aware ranking, we introduce different penalties for players that are similar to the ones previously ranked.

Each set *P*_*i*_ contains a variable number *M*_*i*_ of elements, i.e., $P_{i}=\left\{g_{1},\ldots ,g_{M_{i}}\right\}$, and the sets in F are arbitrarily large and can overlap. We construct a microarray game based on the binary matrix *B* ∈ {0, 1}^*N* × *M*^ where N=|F| and $M=|\cup_{i=1}^{N}P_{i}|$. Each row of *B* represents a set *P*_*i*_ and *B*_*ij*_ = 1 if *g*_*j*_ ∈ *P*_*i*_ while *B*_*ij*_ = 0 if *g*_*j*_ ∉ *P*_*i*_. Each column *i* represents the partial ordering relationship of the element *g*_*j*_ belonging to the set *P*_*i*_.

Given a set $P_{i}\in F$, the Shapley value of *P*_*i*_ is computed following these two steps as proposed in [13]:

1. from the matrix *B*, we get the dictionary A as
  $$\begin{array}{c} A={\left\{\text{sp}\left(B_{j}\right)\right\}}_{j\in M}\subseteq P\left(F\right). \end{array}$$ (3)
  Each set in A represents the support of the corresponding element *g*_*j*_ ∈ *G*.
2. each Shapley value is computed through the formula:
  $$\begin{array}{c} \phi \left(P_{i}\right)=\frac{1}{M}\cdot \sum_{j=1}^{M}(1\left(P_{i}\in \;\text{sp}\left(B_{j}\right)\right)\cdot \frac{1}{\left|\text{sp}(B_{j})\right|}), \end{array}$$ (4)
  where 1 is the standard indicator function returning 1 if the argument is satisfied, i.e., if *P*_*i*_ ∈ sp(*B*_*j*_), and 0 otherwise.

In S1 Appendix, we report a toy example to illustrate the computation of Shapley values in our setup.

Once computed, we can then use these Shapley values to order the sets *P*_*i*_: The higher the Shapley value of a set *P*_*i*_, the more important the set is in the microarray game defined. The importance scores are a measure of the number of elements *g* contained in *P*_*i*_ re-scaled with the size of their supports. If *P*_*i*_ contains elements *g* rarely included in other sets, it will get a higher score. Each *ϕ*(*P*_*i*_) is a real number in [0, 1] and from Shapley values’ properties we know that $\sum_{i=1}^{N}\phi \left(P_{i}\right)=1$. However, the ranking of sets given by the Shapley values alone is unaware of a possible *overlap* among players.

### 3.4 Definition of goals: Redundancy and coverage

Among players in a cooperative game, different kinds of redundancies can appear depending on the structure of the game. When using random variables, redundancy is often measured as the correlation among sets of variables [7]; here, we aim for a redundancy-aware ranking in families of sets. We state that two sets are *redundant* if they share a large number of elements, i.e., if the size of their intersection is large when compared to the size of their union. To measure the redundancy among sets, we use as metric the *Jaccard index J* [37]. Given *A* and *B* two sets, their Jaccard index is $J\left(A,B\right)=\frac{\left|A\cap B\right|}{\left|A\cup B\right|}$. The Jaccard index is a real number in [0, 1] where *J*(*A*, *B*) = 0 if and only if A∩B=⌀ and *J*(*A*, *B*) = 1 if and only if *A* = *B*. Thus, the Jaccard index is direct proportional to the size of the intersection among the sets *A* and *B*.

Having set as a goal the reduction of redundancy within importance scores-based rankings, still we do not want to compromise with the *coverage* of *G*. We hereby define various types of penalties and will compare them w.r.t. coverage *and* redundancy.

1. **Redundancy**—as redundancy measure, we assign to a family of sets *S* the *Jaccard rate* or *Jaccard score* *J*_score_(*S*), i.e.,
  $$\begin{array}{c} J_{\text{score}}\left(S\right)=\frac{1}{\left|S|(|S|-1\right)}\sum_{P_{i},P_{j}\in S,i\neq j}J\left(P_{i},P_{j}\right). \end{array}$$ (5)
  The Jaccard score $J_{\text{score}}\left(S\right)$ represents the average Jaccard index among pairs of sets in *S*; it is a non-negative real number in [0, 1] and $J_{\text{score}}\left(S\right)=0$ if and only if all pairs of sets in *S* do not overlap.
2. **Coverage**—we defined the finite family of sets F and *G* the set of elements in the sets in Section 3.2. Per definition, the family F represents one possible coverage of *G* since each element *g* ∈ *G* is contained at least in one set $P_{i}\in F$. Given a family of sets S⊆P(F), the quantity
  $$\begin{array}{c} c_{G}\left(S\right)=|\cup_{P_{i}\in S}\;P_{i}|\cdot \frac{100}{\left|G\right|} \end{array}$$ (6)
  is the *coverage of G given by S*, i.e., the percentage of elements *g* ∈ *G* that are included at least in one set in *S*. There is an obvious trade-off between the coverage given by *S* and its dimension. In the application to collections of gene sets, we will investigate the success of our methods in preserving the coverage of the entire set while reducing the dimension of F.

Rankings based on Shapley values show a clear tendency to rank bigger sets first. This is reasonable, as the Shapley value counts the number of times the argument of the indicator function is satisfied; Sets with larger sizes are hence the ones for which the indicator function arguments’ are most often satisfied. However, these bigger sets are more likely to overlap as they probably contain elements over-spread through the sets in the family. Hence, when looking for rankings with low redundancy, we will also affect this tendency to rank smaller sets later and bigger ones first.

### 3.5 Different penalties and different rankings

Given the definition of Shapley values as in Eq 4, we obtain importance scores for each of the sets in the family F. We can use these scores to rank the sets in F in a naive manner and refer to this ranking as SV. As mentioned in the previous section, this ranking favors larger sets: a set *P*_*i*_ is contained in a larger number of supports if its size is larger, i.e., the expression $1(P_{i}\in \;\text{sp}\left(g_{j}\right))$ assumes more often value 1 for larger sets. Moreover, it is worth noticing that given two sets *P*_*i*_ and *P*_*j*_ with equal size, the importance score of *P*_*i*_ is larger than that of *P*_*j*_ if *P*_*i*_ contains rarer elements than *P*_*j*_. On the other hand, as the Shapley values tend to rank larger sets first, we expect a high coverage of *G* when selecting even a small number of sets. Moreover, the value function does not include any awareness of overlap among sets (see Section 4) and the ranking SV allocates sets *P*_*i*_, *P*_*j*_ in similar ranking positions when *J*(*P*_*i*_, *P*_*j*_) ≈ 1.

We introduce various penalties in order to penalize overlapping sets and not rank them similarly. The introduced penalties are functions of the Jaccard index among sets, such that pairs of subsequently ranked sets are characterized by low Jaccard rates. We provide a detailed comparison of the obtained rankings in Section 4. Using the application to collections of gene sets, we illustrate the properties of each of the obtained rankings and how to select the most useful for the purpose at hand. An overview of the proposed methods highlights that

- the proposed penalties are rather flexible and can be adapted to optimize specific properties, and
- there is not a perfect and unique choice fulfilling all goals.

**Fig 1:**
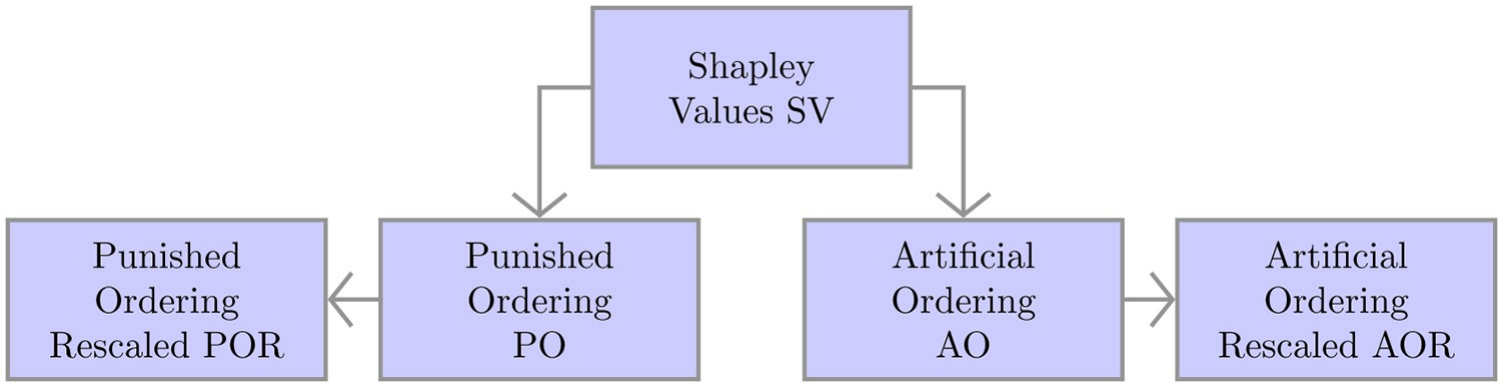
Genealogical tree of the penalties’ criteria.

The obtained rankings are constructed one on top of the other as represented in Fig 1. Moreover, the rankings are constructed using a greedy approach that selects one set at a time; as the computation of the Shapley values using the constructed microarray game is not computationally expensive, this still leads to feasible run-times for the entire ranking.

1. **Penalized Ordering PO**—the Shapley values are used to obtain the first ranked set ${\tilde{P}}_{1}$, i.e., the one whose Shapley value is the highest.
  In the second step, all Shapley values are re-computed for the not-yet ranked sets and $J({\tilde{P}}_{1},P)$ is subtracted from them, i.e., the Jaccard index among ${\tilde{P}}_{1}$ and the to-be-ranked set *P*. For each set $P\neq \tilde{P_{1}}$, the importance score *S*_2_(*P*) at the second step reads
  $$\begin{array}{c} S_{2}\left(P\right)=\phi_{2}\left(P\right)-J\left({\tilde{P}}_{1},P\right). \end{array}$$ (7)
  The penalty being the Jaccard index aims to penalize sets that are highly overlapping with ${\tilde{P}}_{1}$. The set arg max_*P*_ *S*_2_(*P*) obtains the second position and the process restarts. The penalty is automatically increased in each step: in particular, after selecting the first *n* sets, the score *S*_*n*+1_(*P*) obtained by the set *P* (where *P* has not been ranked yet at step *n* + 1) is given by the following recursive formula
  $$\begin{array}{c} \begin{array}{ccc} S_{1}\left(P\right) & = & \phi_{1}\left(P\right)\\ S_{n+1}\left(P\right) & = & \phi_{n+1}\left(P\right)-\sum_{i=1}^{n}J\left(\text{arg}\;\underset{\bar{P}}{\text{max}}S_{i}\left(\bar{P}\right),P\right),\;\text{if}\;n\geq 1 \end{array} \end{array}$$
  and, at the step *n* + 1, the algorithm ranks the set ${\tilde{P}}_{n+1}=\text{arg}\;{\text{max}}_{P}\;S_{n+1}\left(P\right).$
  We underline that the Shapley values are re-computed after each iteration and *ϕ*_*n*_(⋅) represents the Shapley value function at iteration *n*. The re-computation is necessary for two main reasons: first, to satisfy the efficiency property, i.e., $\sum_{P_{i}}\phi_{m}\left(P_{i}\right)=1$ for each *m* where the sum is computed over the sets which have not been ranked yet; Second, the set A changes at each iteration, implying a (possible) different order of the sets when the Shapley values are re-computed.
2. **Penalized Ordering with Rescaling POR**—POR ranking adds to PO a rescaling of the penalty; the ordering obtained using PO automatically increases the penalty after each iteration. After a sufficiently large number of iterations, this process can lead to penalties larger than the Shapley values themselves and sets end up being assigned negative importance scores. Two major issues are connected with this: (1) negative importance scores are hardly interpretable and (2) the penalties can become too harsh with respect to the Shapley values. Thus, we propose to re-scale the penalty: in POR, the term $\sum_{i=1}^{n}J\left(\text{arg}\;{\text{max}}_{\bar{P}}\;S_{i}\left(\bar{P}\right),P\right)$ is re-scaled to the interval [0, max_*P*_{*ϕ*_*i*_(*P*)}] at each iteration.
3. **Artificial Ordering AO**—the introduction of an artificial set, represents our attempt to avoid penalizing each set multiple times for containing the same elements. The artificial set *AP*_*n*_ is updated in each iteration *n*. It is initialized at step 1 to $AP_{1}={\text{arg}\;\text{max}}_{P}\;\phi_{1}\left(P\right)$. At iteration *n*, *AP*_*n*_ is updated with the elements of the last ranked set
  $$\begin{array}{c} AP_{n}=\cup_{i=0}^{n}\underset{P}{\text{arg}\;\text{max}}\;S_{i}\left(P\right). \end{array}$$
  The importance score is defined as in PO, but penalizing with a unique Jaccard index with the set *AP*_*n*_ instead of using the sum of Jaccard indeces with the sets previously ranked. In AO the penalty depends on the elements in *G* that the first *n* ranked sets have covered. This avoids multiple penalties for the same overlapping elements; thus the penalties will be *softer* with respect to these.
4. **Artificial Ordering with Rescaling AOR**—as in the re-sacled version of PO, the re-scaling is done for AO on the term *J*(*AP*_*n*_, *P*) to the interval [0, max_*P*_{*ϕ*_*i*_(*P*)}]. The aim here is again avoiding too harsh penalties eventually causing negative importance scores.

The SV ranking is not functional for the purposes of both maximizing the importance scores given by the Shapley values of the microarray game and lowering the redundancy among the subsequent pairs of ranked sets. Thus, we introduced four different penalties for the construction of final rankings. The two penalized rankings, PO and POR, consider only overlaps among pairs of sets. The penalty is increased at each step by the addition of the Jaccard rate among the last pair of sets; thus if there are elements contained in multiple sets of the family, these elements will affect the penalty terms multiple times. As this might be problematic in the long run for small sets containing some of these often-appearing elements, we introduced the artificial set AP to create the rankings AO and AOR. Using the artificial pathways, we solve the problem of multiple punishments as the overlaps with single elements are penalized exactly once. In both AOR and POR, the re-scalings attenuate the effect of the penalties, such that the scores are kept positive also for the sets whose penalties are higher than the Shapley values themselves. Note that each penalty still orders the set with the highest Shapley value first. The orderings start to differ from each other from the second-ranked set on.

We include the pseudo-code for reproducibility reasons while the implemented code is publicly available at Github. In S1 Appendix, we provide additional details on the construction of the penalties.

**Algorithm 1** Penalized Shapley Values

1: **procedure** (F, criteria, re-scaled = FALSE)

2:  take as input the family of sets F

3:  ${\tilde{P}}_{1}=\text{arg}\;{\text{max}}_{P}\left\{\phi_{1}\left(P\right)\right\}$

4:  $LS={\tilde{P}}_{1}$

5:  $F=F\backslash {\tilde{P}}_{1}$

6:  initialize penalty *to* 0

7:  **for** i∈range(1,|F|+1) **do**

8:   $AP_{i}=\cup_{k<i}\;{\tilde{P}}_{k}$

9:   **for** P∈F **do**

10:    **if** criteria == PO **then**

11:     penalized(*P*) = penalized(*P*)+ *J*(*LS*, *P*)

12:     **if** re-scaled == TRUE **then**

13:      $\text{penalized}\left(P\right)=\text{penalized}\left(P\right)\cdot \frac{{\text{max}}_{k}\left\{\phi_{i}\left(P_{k}\right)\right\}}{{\text{max}}_{k}\{\text{penalized}}\left(P_{k}\right)\}$

14:     *S*_*i*_(*P*) = *ϕ*_*i*_(*P*) − penalized(*P*)

15:    **else if** criteria == APO **then**

16:     penalized(*P*) = *J*(*AP*_*i*_, *P*)

17:     **if** rescaled == TRUE **then**

18:      $\text{penalized}\left(P\right)=\text{penalized}\left(P\right)\cdot \frac{{\text{max}}_{k}\left\{\phi_{i}\left(P_{k}\right)\right\}}{{\text{max}}_{k}\{\text{penalized}}\left(P_{k}\right)\}$

19:     *S*_*i*_(*P*) = *ϕ*_*i*_(*P*) − penalized(*P*)

20:   ${\tilde{P}}_{i}=\text{arg}\;{\text{max}}_{P}\left\{S_{i}\left(P\right)\right\}$

21:   $LS={\tilde{P}}_{i}$

22:   $F=F\backslash {\tilde{P}}_{i}$

23:   *i* + +

**return** ordering $\tilde{P}$

## 4 Results: Ranking pathways in collections of gene sets

Implementing our game-theoretic concept provides a new framework to reduce the dimension of families of sets. The obtained rankings are used to select the first *n* ranked sets, allowing for a lower overlap among sets, high coverage of the elements with a lower number of sets, thus increasing interpretability of the families of sets. Moreover, in contrast to previously introduced methods, our approach is not altering the sets.

Collections of gene sets are a promising application of our rankings and the source of inspiration for the proposed method. In particular, the collections of gene sets F are sets of pathways, and pathways *P*_*i*_ are sets of genes *g*_*j*_; hence, the collections of gene sets are families of sets, and the pathways are the sets to be ranked.

We use this application to illustrate that the proposed rankings:

- provide a ranking of the original pathways in the collections of gene sets without modifying them;
- *reduce the redundancy* among subsequently ranked pathways;
- maintain a *high coverage* of the genes in the collection of gene sets when selecting the first *n* ranked pathways;
- do not favor larger gene sets;
- reduce the size of gene sets collections, thus increasing interpretability.

The experiments emphasize that the choice of which particular penalty to use is highly dependent on the goal, and there is not a *unique correct* way of choosing which ranking to use.

In Table 1 we summarize the properties of the different rankings based on the analysis of different data sets. We present the results for four collections of gene sets, i.e., the KEGG, CGN, CM, and TFT LEGACY. To complete our analysis, we investigate the effects of the different penalties and descending pathways selections on the gene set enrichment analysis looking for the significance of pathways for different association traits. Section 6 contains the details about the data availability and the necessary links.

**Table 1:** Comparison between the original SV and the newly proposed rankings.

	correlation with pathways’ sizes	redundancy	coverage
**SV**	positive correlation	reference level	reference level
**PO**	negative correlation	much less	same
**POR**	no correlation	less	less
**AO**	negative correlation	much less	same
**AOR**	no correlation	less	less

In Table 3 in S1 Appendix, we included additional analyses comparing our game theoretic approach also with more classical enrichment analysis methods. There we also focused on the ability to detect significant pathways after reducing the gene set, with respect to 38 phenotypic traits.

Given that we present arbitrary choices both for collections of gene sets and phenotypic traits, our experiments and analyses should be seen as exploratory in the spirit of illustrative case studies. We explicitly do not claim the generalizability of these results.

### 4.1 Correlation with the size of pathways

The Shapley value function assigns to a set P∈F a positive real number incorporating information on the distribution of its elements in the other sets of F. This leads to a positive correlation with the size of pathways, i.e., larger sets are more likely to get a higher Shapley value. In Table 2, Kendall’s *τ* scores measure the ordinal association between the size of pathways and their position in the rankings. The table clearly displays that when ranking the pathways using SV, we tend to rank larger pathways first. Using AO and PO, this effect is reversed in most collections of gene sets; in particular, AO and PO rankings select small pathways first while larger ones are ranked last in the orderings. In AOR and POR, there is no clear tendency of a correlation between the dimension of pathways and the position in the ranking.

**Table 2:** Correlation among size and position in the rankings. Kendall’s *τ* coefficients measuring the correlation among the position in the ranking and the size of the gene sets.

	SV	PO	POR	AO	AOR
**KEGG**	0.49	-0.21	0.15	0.23	0.19
**CGN**	0.43	-0.51	0.019	-0.702	-0.041
**CM**	0.759	-0.531	0.019	-0.702	-0.041
**TFT LEGACY**	0.679	-0.460	0.354	-0.828	0.458

Our goal was to reduce the redundancy among subsequently ranked pathways. Hence, we indirectly affect the strength of the correlation between ranking position and size as larger pathways are more likely to show overlapping among them. The rankings SV, PO, and AO, show similar behaviors across the different studied collections of gene sets, while the re-scaled penalties show no clear tendency.

### 4.2 Redundancy awareness

**Fig 2:**
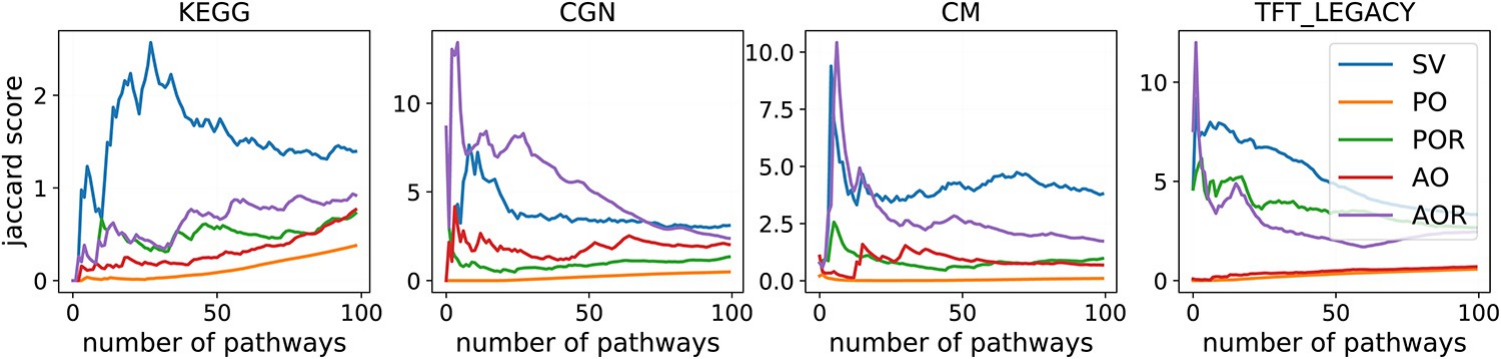
Redundancy awareness. In the plots: average re-scaled Jaccard scores of sets of pathways ranked up to *j*-th position (*x* axis). In each of the collection of gene sets, we select up to 100 pathways. In the table: re-scaled Jaccard rates of the first 10, 20 and 40% of the gene sets; In each column, underlined text indicates the minimum Jaccard score.

The introduced penalties ensure rankings that take into account the overlap among subsequent ranked gene sets. We evaluate the redundancy using the Jaccard score as defined in Eq (5). To get comparable numbers throughout different collections of gene sets, we re-scale the Jaccard scores to the *maximum Jaccard score*, i.e., the maximum Jaccard index among any pairs of pathways within the collection of gene sets. In Fig 2, we plot the re-scaled Jaccard scores as a function of the number of pathways. We first compute the rankings, then select the respective first *n* ranked gene sets and compute the Jaccard rate of the obtained set. The lower the Jaccard rate, the better the ranking performs in selecting non-overlapping pathways.

The table in Fig 2 shows the re-scaled Jaccard rates for some reference values (10, 20 and 40% of the pathways). The ranking achieved the lowest Jaccard rates is PO; AO performed well in (almost) all collections of gene sets. PO and AO use strong penalties such that highly overlapping pathways were ranked far from each other. Moreover, we note that the classical SV ordering performed the worst in all but one case as it is not aware of redundancies.

### 4.3 Coverage of gene sets

**Fig 3:**
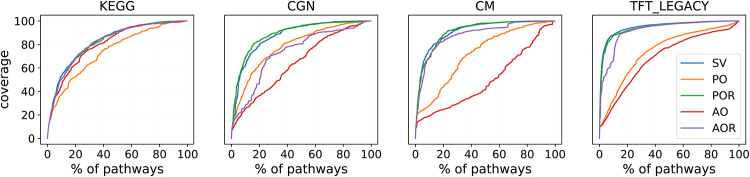
Cumulative coverage of gene sets. In the plots: the coverage of the genes as a function of the number of pathways included using the different rankings. In the Table: coverage of the genes when selecting respectively the 10, 20 and 40% of the pathways. In each column, underlined text highlights the highest coverage.

We investigate the ability of our methods to cover the genes using a limited amount of pathways. In Fig 3, we plot the coverage of the genes in percentage when only considering the first *n* ranked pathways. The SV ranking gets a generally high coverage of genes in the collections of gene sets. We note that the orderings SV, POR, and AOR are clearly outperforming the rankings given by PO and AO.

Moreover, we compare the different rankings using some reference levels: In particular, the table in Fig 3 gives insights into the proportions of the genes that can be covered using only a limited percentage of pathways (10, 20 and 40% of the pathways). The high coverage achieved by SV is due to the correlation between the size of the pathways and their positions in the ranking; however, not the same can be argued about POR and AOR as we demonstrated that the correlation with the gene sets’ sizes had been reduced. Maximizing Shapley values while minimizing redundancy achieves outstanding performances in both cases. The lower performances of PO and AO are explained by the penalties, which are generally harsh for overlapping gene sets; Hence, they select first small pathways ranked in the lowest positions by Shapley values alone, as already argued (cf. Table 2).

On the other hand, we observe that the rankings do not outperform the original SV ordering in covering the entire gene set; the advantages of the penalized orderings are evident when considering that the performances of the newly proposed rankings are close to the original SV ranking while retaining a much smaller amount of redundancy and not preferring large pathways.

### 4.4 Number of significant pathways

Finally, we investigate how the proposed rankings relate to gene set enrichment analysis when using only the first *n* ranked pathways. We use Fisher’s exact test [38, 39] to determine whether a pathway is significant or not and apply multiple hypothesis testing corrections for the p-values (Bonferroni or FDR correction [26, 31]).

Using the proposed method of ranking and selecting, we obtain smaller collections of gene sets. Afterwards, we test for associations with specific phenotypic traits and compare the number of significant pathways found in the original collections of gene sets and in the reduced ones.

**Fig 4:**
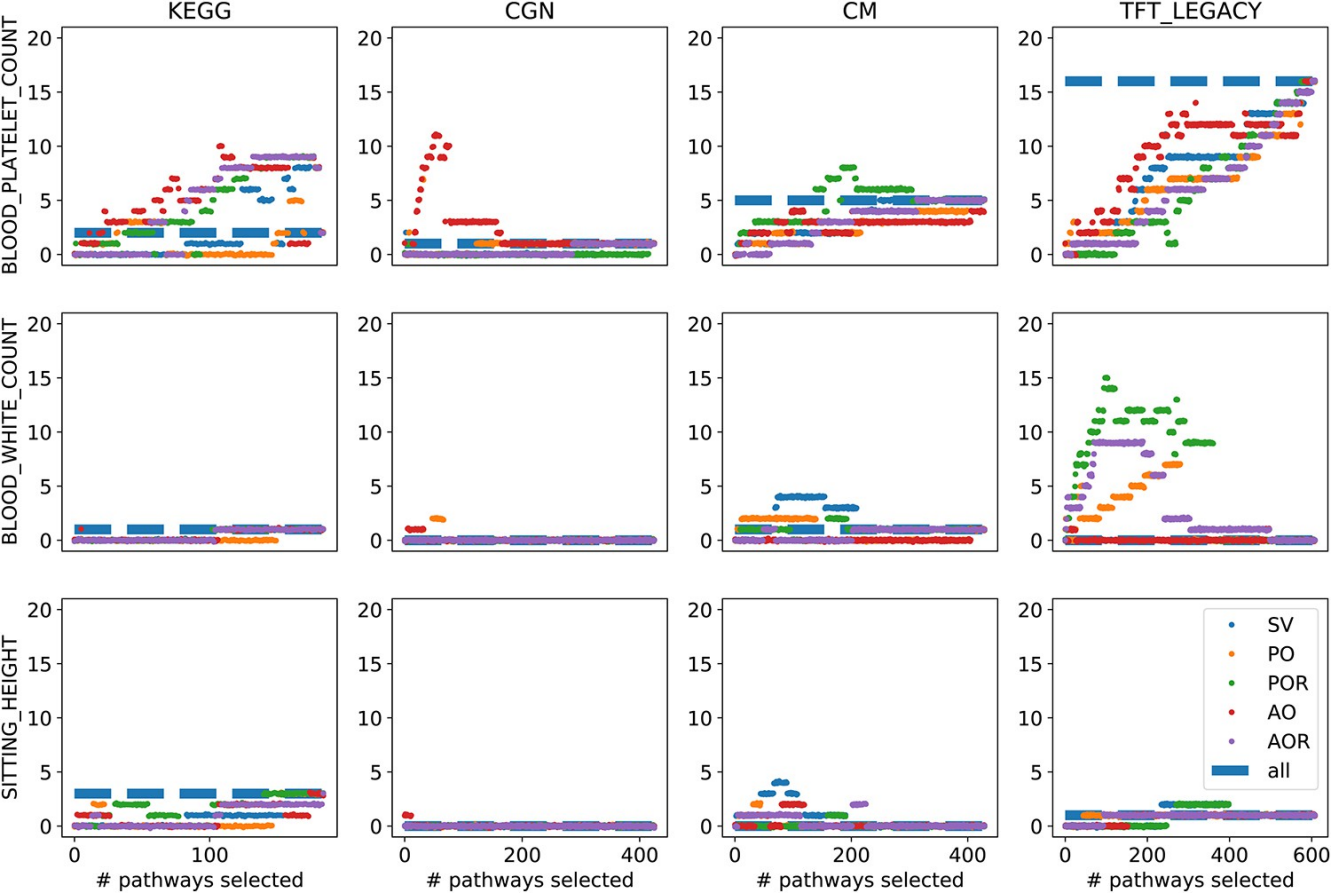
Significant pathways detected. We show results on three selected association traits for the four collections of gene sets. In each plot, the *x*-axis represents the number of pathways included in the multiple statistical testing while the *y*-axis represents the number of statistical significant pathways found. The different colours represent the different proposed orderings as in the legend. We used FDR correction for multiple testing and *α* = 0.05.

Fig 4 illustrates for each collection of gene sets the number of statistically significant pathways founds for some association traits, i.e., *blood platelet count*, *blood white count* and *sitting height*. The plots refer to the FDR correction for multiple hypotheses testing. For sensitivity reasons, we additionally include in Table 3 in S1 Appendix a comparison among the number of significant pathways found when using (1) the first 40% of ranked pathways from the collection of gene sets KEGG2013 and BioCarta2015 with the proposed methods, (2) the complete collection of gene sets using Fisher Exact test and adjusting p-values using the FDR correction and (3) the Enrich method; in the comparison, we used significant level *α* = 0.05 and 38 phenotypic traits from the TWAS hub.

The number of significant pathways in each collection of gene sets is represented in each plot as a blue dashed line. We observe that the number of significant pathways found when limiting the number of tested pathways using the introduced rankings highly depends on the collection of gene sets and the particular trait. In some settings using only a limited number of pathways may lead to a higher number of pathways reaching significance, while in other settings, we detected a lower number of significant pathways. This happens when significantly associated pathways are not ranked among the first *n*, which obviously can occur when applying our unsupervised feature selection techniques.

In conclusion, the number of significant pathways discovered when using the proposed rankings remains, on average, the same as using the whole collections of gene sets. Reducing the collections of gene sets to a limited amount of pathways using unsupervised approaches like the one we propose might lead to a better interpretability and better handling of gene sets, but not necessary to a higher statistical power in enrichment analyses. Whether the number of significant gene sets found is increasing or decreasing highly depends on the phenotypic trait and the collection of gene sets used.

## 5 Discussion

Shapley values are often used to assign a fair value to players based on their contribution to the game. However, they ignore eventual redundancies among players, which hinders their performance and can bias the resulting scores towards redundant players.

In this work, we proposed a game-theoretical approach to incorporate redundancy-awareness into Shapley values to rank gene sets in an unsupervised fashion. In particular, we proposed different ways to penalize the overlapping sets so they are not progressively selected.

We studied four different penalties, and we were able to show that the orderings obtained are

- *not favoring larger players*—applying the redundancy-aware penalties avoids that larger gene sets are ranked first;
- *redundancy free*—the combination of Shapley values with the redundancy reduction criteria shows high effectiveness in maintaining the importance of sets given by Shapley values while reducing the redundancy among the first ranked pathways;
- *achieving high coverage*—the obtained rankings still lead to high coverage of genes. We showed that a positive correlation with the size of sets is not the unique solution in order to achieve high coverage of the genes, i.e., the original Shapley values ranking is not performing much better than the orderings which rank first small sets keeping low redundancy rates;
- *on average do not have a high influence on the number of detected significant pathways*—having fixed the collection of gene sets, the number of significant pathways detected when applying GSEA techniques with multiple hypotheses testing corrections increases and decreases compared to the full gene set, depending on the phenotype.

### 5.1 Comparison among rankings

In light of the results in Figs 2 and 3, we conclude that AO leads to the least favorable ranking in covering the genes while the two re-scaled orderings together with SV are the best-performing ones regarding coverage. Regarding the reduction of redundancy among pathways, all penalized orderings can achieve this goal by outperforming the SV ranking. Lastly, when comparing the different methods with respect to the correlation with the size of pathways, we see that only AOR and POR do not lead to any specific correlation.

Hence, we conclude that POR is the best ordering one could choose when the aim is to optimize the ranking for redundancy elimination and coverage of the genes without incurring specific correlations with the gene sets’ sizes.

### 5.2 Limitations and future work

Our results suggest that using our tool as a pre-processing step for collections of gene sets, we get on average similar numbers of significant pathways when checking for association with phenotypic traits (although relying on much fewer pathways). In other words, we evaluated the significance of the top-ranked pathways with respect to phenotypic traits yielding very similar results to the unfiltered collections of gene sets. In some cases, however, we observe fewer significant pathways than considering the whole collections. Therefore, if the interest was on increasing the statistical power on specific collections of gene sets and phenotypic traits, using a supervised method should be used to reduce the number of pathways. Our rankings based on Shapley values are unsupervised, based on the structure of the collections of gene sets and pathways and their overlaps; thus, they are not meant to increase the number of significant pathways detected in supervised contexts.

Starting from our work, further research could focus on how to increase the statistical power for specific collections of gene sets and traits: One possible extension is the addition of a supervised term to the penalty that also considers the relevance of single pathways to specific phenotypic traits. This could potentially lead to a higher number of significant pathways when using the Shapley values’ based rankings to reduce the dimension of the collections of gene sets.

The proposed ranking methods can be applied in any family of sets or where the relationship can be expressed as a binary matrix *B*; therefore, the possible applications of the proposed techniques are broad and various. From the theoretical point of view, further research is warranted on understanding and creating a comprehensive theory on the redundancy among players and how to deal with the redundancy unawareness of Shapley values.

## 6 Conclusions

We proposed the first redundancy-aware Shapley values to rank sets in families of sets. The four presented rankings aim to satisfy various properties when selecting sets based on them. Motivated by the numerous applications of unsupervised feature selection, the proposed importance scores consider the distribution of elements within the family of sets and their overlap. In the presented application of the new methods to rank pathways in collections of gene sets, they performed well with respect to various metrics. However, one can assume that the range of potential applications is much broader. We hence have reason to believe that our proposed methods can open new research paths both in the applied and theoretical fields.

## Supporting information

S1 Appendix (PDF)
