# Supplementary material for: Redundancy-aware unsupervised ranking based on game theory: Ranking pathways in collections of gene sets
Source: PLoS One. 2023 Mar 9;18(3):e0282699. doi: 10.1371/journal.pone.0282699 (PMC9997904; doi:10.1371/journal.pone.0282699)
Supplement: S1 Appendix — (PDF) [file pone.0282699.s001.pdf]

A Sum-Of-Unanimity Games (SOUG)

Consider a set of players $\mathcal{N}$ and a coalition $T \subseteq \mathcal{N}$; the associated *unanimity game* u_T is defined by

$$u_T(S) = \begin{cases} 1 & \text{if } S \subseteq T \\ 0 & \text{otherwise} \end{cases} \quad \text{for any } S \subseteq \mathcal{N}$$

It can be proved that given any cooperative game $(\mathcal{N}, v)$, the value function v can be written as the linear combination of Unanimity Games in a unique way, i.e.,

$$v(\cdot) = \sum_{T \in \mathcal{P}(\mathcal{N})} \lambda_T(v) u_T(\cdot),$$

where $\lambda_T(v) \in \mathbb{R}$ are called *unanimity coefficients* and are determined by the formula

$$\lambda_T(v) = \sum_{S \in \mathcal{P}(\mathcal{N})} (-1)^{t-s} v(S)$$

As we see, the computation of $\lambda_T(v)$, as well as the one of $\phi_v(i)$ becomes intractable if N increases.

The SOUG allow for polynomial time computation of Shapley values. In particular, the computation in terms of the unaminity coefficients $\lambda_T(v)$ is reduced to

$$\phi_v(i) = \sum_{T \subseteq \mathcal{N} \setminus \{i\}} \frac{\lambda_T(v)}{|T|}$$

for each player i in $\mathcal{N}$.

It can be proven that any cooperative game $(\mathcal{N}, v)$ has a unique formulation as a sum of unanimity games. However, finding the equivalent SOUG of a game $(\mathcal{N}, v)$ is computationally equivalently hard as computing the Shapley values.

Using SOUG brings the essential advantage of polynomial run-time when dealing with big families of sets, e.g., gene sets and pathways.

B Glove game

A classical example of a cooperative game is the so-called *glove game*. Consider the set of players $\{A, B, C\}$; A and B are right-hand gloves while C is a left-hand glove. A coalition, i.e., a subset of $\{A, B, C\}$, has value 1 if it contains a pair of gloves (left + right) and has value 0 if it does not. A person is wearing one pair of gloves at a time, therefore adding more gloves to a coalition already containing a pair of gloves is useless; we represent this mathematically - any coalition containing a pair does not increase its worth when including more gloves. The *grand coalition* $\{A, B, C\}$ contains one pair of gloves, i.e., the pair $\{A, C\}$ or the pair $\{B, C\}$, therefore it has value equals to 1. Note that the value function assigns 1 to the grand coalition and 0 to the empty set. After computing the Shapley values, we find $\phi(A) = \phi(B) = \frac{1}{6}$ and $\phi(C) = \frac{1}{3}$. Players A and B get the same Shapley values as they are essentially indistinguishable. Shapley values scores do not detect the existing *redundancy* among A and B. After including one element among A and B, including the other does not yield any advantages. We refer to this similarity among players as *redundancy* and we say that the Shapley values are unaware of redundancy among players.

C Example of computation of Shapley values

We give here an example on how to compute the Shapley values in a toy example. Consider a family of sets

$$\mathcal{F} = \{P_1 = \{a, b, c, e\}, P_2 = \{a, d\}, P_3 = \{c, d, e\}\}.$$

The union of the sets in $\mathcal{F}$ is $G = \{a, b, c, d, e\}$. We construct the binary matrix B, i.e.,

$$B = \begin{pmatrix} 1 & 1 & 1 & 0 & 1 \\ 1 & 0 & 0 & 1 & 0 \\ 0 & 0 & 1 & 1 & 1 \end{pmatrix}$$

where each column represents an elements of G, respectively a, b, c, d, e and the zeroes and ones components of B represent the binary relationship of being included in each set of $\mathcal{F}$. We then get the dictionary $\mathcal{A}$ as described in Equation (3) in Section 3.3. From the first column of B follows that a belongs to P_1 and P_2 but not to P_3 thus the set $\{P_1, P_2\}$ is the firstset in $\mathcal{A}$. After applying the same procedure on each column of B, we get

$$\mathcal{A} = \{\{P_1, P_2\}, \{P_1\}, \{P_1, P_3\}, \{P_2, P_3\}, \{P_1, P_3\}\}.$$

Now, we calculate the Shapley values as in Equation (4), leading to

$$\begin{aligned}
\phi(P_1) &= \frac{1}{5} \cdot \left(1 \cdot \frac{1}{2} + 1 \cdot 1 + 1 \cdot \frac{1}{2} + 0 \cdot \frac{1}{2} + 1 \cdot \frac{1}{2}\right) = \frac{1}{2} \\
\phi(P_2) &= \frac{1}{5} \cdot \left(1 \cdot \frac{1}{2} + 0 \cdot 1 + 0 \cdot \frac{1}{2} + 1 \cdot \frac{1}{2} + 0 \cdot \frac{1}{2}\right) = \frac{1}{5} \\
\phi(P_3) &= \frac{1}{5} \cdot \left(0 \cdot \frac{1}{2} + 0 \cdot 1 + 1 \cdot \frac{1}{2} + 1 \cdot \frac{1}{2} + 1 \cdot \frac{1}{2}\right) = \frac{3}{10}
\end{aligned}$$

As expected, we observe that the sum of Shapley values equals 1. Moreover, we can notice that there is a bias towards sets that have higher dimensions, i.e., the ordering P_1, P_3 and P_2 reflects also the ordering of the sets with respect to their sizes. This example illustrates that Shapley values do not aim for low intersection among ranked sets or high coverage.

The sets in $\mathcal{F}$ are ordered according to Shapley values as P_1, P_3 and P_2. However P_1 and P_3 share two elements while P_1 and P_2 share only one. If we want to select two out of these three subsets in order to maximize the coverage of G while keeping low the overlapping among the sets, we would better select P_1 and P_2 instead of P_1 and P_3.

D Different rankings

We introduced in the paper four different orderings which depend on different penalization criteria. The difference among the penalization criteria is reflected in the coverage and redundancy reduction performances of the various orderings. We give here detailed information on the algorithm for sake of completeness and for reproducibility purposes.

The Shapley values need to be re-computed after each iteration as, after the selection of some pathways, they do not sum anymore to 1. Moreover, the removal of one set can change the ordering of the other sets as the Shapley values depend on the distribution of the elements among the sets.

We give here more details on the construction of the various penalized rankings in the context of pathways' orderings. The details here introduced are true in general in any family of sets.

- **Penalized Ordering (PO)** – The penalty grows at each step as we add it to the Jaccard index with the last ranked pathway: the score $S_{n+1}(P)$ obtained by the pathway P (where P has not been ranked yet at step $n+1$) is given by the following iterative formula

$$\begin{array}{lcl} S_1(P) & = & \phi_1(P) \\ S_{n+1}(P) & = & \phi_{n+1}(P) - \sum_{i=1}^{n} J(\arg\max_{\bar{P}} S_i(\bar{P}), P), \qquad \text{if } n \geq 1 \end{array}$$

  and, at the step $n+1$, the algorithm ranks the set $\tilde{P}_{n+1} = \arg\max_P S_{n+1}(P)$. $\phi_n(\cdot)$ represents the Shapley value function at the nth iteration.

- **Penalized Ordering Re-scaled (POR)** – The algorithm shows strong similarities with PO. The only difference relies in the penalty which is re-scaled in POR to the interval $[0, \max_P\{\phi_i(P)\}]$. This avoids that, after the first n steps, the highest importance scores assume negative real numbers as the penalties of PO can assume values higher than 1.

  The importance scores are defined as follows:

$$\begin{array}{lcl} S_1(P) & = & \phi_1(P) \\ R_n & = & \frac{\max_P \phi_n(P)}{\max_P \sum_{i=1}^{n} J}(\arg\max_{\bar{P}} S_i(\bar{P}), P) \\ S_{n+1}(P) & = & \phi_{n+1}(P) - \sum_{i=1}^{n} J(\arg\max_{\bar{P}} S_i(\bar{P}), P) \cdot R_n, \qquad \text{if } n \geq 1. \end{array}$$

  The complexity of the algorithm proposed does not change.

- **Artificial Ordering (AO)** – After ranking the first pathway, it computes an *artificial pathway* which includes all genes belonging at least to one of the pathways previously selected.

  In order to rank the $(n+1)$-th set, PO (and POR) penalizes multiple times the overlap among sets that share some elements g. The introduction of the artificial gene set AP_n i.e.,

$$AP_n = \cup_{i=0}^{n} \arg\max_P S_i(P).$$

  avoids these multiple penalties. Then the scores are similarly computed as

$$\begin{array}{lcl} S_1(P) & = & \phi_1(P) \\ S_{n+1}(P) & = & \phi_{n+1}(P) - J(AP_n, P), \qquad \text{if } n \geq 1. \end{array}$$

- **Artificial Ordering Re-scaled (AOR)** – The difference with the AO ordering is the re-scaling of the penalty to the interval $[0, \max_P\{\phi_i(P)\}]$.

$$\begin{array}{lcl} S_1(P) & = & \phi_1(P) \\ R_n & = & \frac{\max_P \phi_n(P)}{\max_P J}(AP_n, P) \\ S_{n+1}(P) & = & \phi_{n+1}(P) - J(AP_n, P_j) \cdot R_n, \qquad \text{if } n \geq 1. \end{array}$$

  As in POR, the complexity of the algorithm proposed does not change.

		HEIGHT	BLOOD PLATELET COUNT	STANDING HEIGHT	BLOOD RED COUNT	HEEL TSCORE	BLOOD WHITE COUNT	BLOOD EOSINOPHIL COUNT	SITTING HEIGHT	TRUNK FAT FREE MASS	TRUNK PREDICTED MASS	WHOLE BODY FAT FREE MASS	WHOLE BODY WATER MASS	SYSTOLIC BLOOD PRESSURE	BASAL METABOLIC RATE	IMPENDANCE WHOLE BODY	BMI	COMP. HEIGHT SIZE AGE 10	ARM PREDICTED MASS R	ARM FAT FREE MASS R	LEG FAT FREE MASS R	LEG PREDICTED MASS R	LUNG FEV1FVC RATIO	IMPENDANCE LEG R	IMPENDANCE ARM R	LUNG FVC	WEIGHT	WHRATIO	HAIR PIGMENT	HIP CIRCUMFERENCE	TRUNK FAT MASS	WHOLE BODY FAT MASS	ARM FAT MASS R	LEG FAT MASS R	TRUNK FAT PERCENTAGE	BODY FAT PERCENTAGE	ARM FAT PERCENTAGE R	LEG FAT PERCENTAGE R	CARDIOVASCULAR DISEASE
KEGG13	SV		-	-	-	-	-	-	-	-	-	-	-	-	-	-	-	-	-	-	-	-	-	-	-	-	-	-	-	-	-	-	-	-	-	-	-	-	-
	PO	7	-	-	-	-	-	-	-	-	-	-	-	-	-	-	-	-	-	-	-	-	-	-	-	-	-	-	-	-	-	-	-	-	-	-	-	-	-
	POR	-	-	-	1	3	-	-	-	-	-	-	-	-	-	-	-	-	-	-	-	-	-	-	-	-	-	-	-	-	-	-	-	-	-	-	-	-	-
	AO	7	1	-	1	-	-	-	-	-	8	-	-	-	-	-	-	-	-	-	-	-	-	-	-	-	-	-	-	-	-	-	-	-	-	-	-	-	-
	AOR	7	-	3	-	-	-	-	-	-	8	-	-	-	-	-	-	-	-	-	-	-	-	-	-	-	-	-	-	-	-	-	-	-	-	-	-	-	-
	ALL	7	1	3	1	3	1	-	-	1	8	-	-	-	-	-	-	-	-	-	-	-	-	-	-	-	-	-	-	-	-	-	-	-	-	-	-	-	-
	ENR	-	-	-	-	4	-	-	-	-	-	-	-	-	-	-	-	-	-	-	-	-	-	-	-	-	-	-	-	-	-	-	-	-	1	-	-	-	-
BIOCARTA	SV	-	-	-	-	-	-	-	-	-	-	-	-	-	-	-	-	-	-	-	-	-	-	-	-	-	-	-	-	-	-	-	-	-	-	-	-	-	-
	PO	2	-	2	-	-	-	-	-	-	1	-	-	-	-	-	-	-	-	-	-	-	-	-	-	-	-	-	-	-	-	-	-	-	-	-	-	-	-
	POR	2	-	2	2	-	-	-	-	-	-	-	-	-	-	-	-	-	-	-	-	-	-	-	-	-	-	-	-	-	-	-	-	-	-	-	-	-	-
	AO	2	-	2	2	-	-	-	-	-	1	-	-	-	-	-	-	-	-	-	-	-	-	-	-	-	-	-	-	-	-	-	-	-	-	-	-	-	-
	AOR	-	-	-	2	-	-	-	-	-	-	-	-	-	-	-	-	-	-	-	-	-	-	-	-	-	-	-	-	-	-	-	-	-	-	-	-	-	-
	ALL	2	-	2	2	1	-	-	-	-	1	-	-	-	-	-	-	-	-	-	-	-	-	-	-	-	-	-	-	-	-	-	-	-	-	-	-	-	-
	ENR	2	-	-	11	1	-	3	-	-	-	-	1	-	1	-	-	-	-	-	-	-	-	-	-	-	-	-	-	-	1	1	-	-	-	-	-	-	2

Table 3. Comparison of the number of significant pathways. Number of significant pathways found using Fisher Exact Test and FDR correction when using the 40% of the pathways in the collection of gene sets ranked using the proposed orderings (namely, SV, PO, POR, AO and AOR) and the complete collection of gene sets (ALL); the ENR row contains the number of significant pathways for each of the phenotypic traits using the Enrich method.
